# Clinical and biochemical footprints of inherited metabolic diseases. XIII. Respiratory manifestations

**DOI:** 10.1016/j.ymgme.2023.107655

**Published:** 2023-07-24

**Authors:** Alessandro Rossi, Simona Basilicata, Melissa Borrelli, Carlos R. Ferreira, Nenad Blau, Francesca Santamaria

**Affiliations:** a Department of Translational Medical Sciences, University of Naples Federico II, Naples, Italy; b National Human Genome Research Institute, National Institutes of Health, Bethesda, MD, USA; c Division of Metabolism, University Children’s Hospital, Zürich, Switzerland

**Keywords:** Inherited metabolic diseases, Respiratory manifestations, Lung disease, Interstitial lung disease, Sleep disordered breathing, Upper airways obstruction

## Abstract

At any age, respiratory manifestations are a major cause of increased morbidity and mortality of inherited metabolic diseases (IMDs). Type and severity are extremely variable, this depending on the type of the underlying disorder. Symptoms and signs originating from upper or lower airways and/or thoracic wall and/or respiratory muscles involvement can occur either at presentation or in the late clinical course. Acute respiratory symptoms can trigger metabolic decompensation which, in turn, makes airway symptoms worse, creating a vicious circle. We have identified 181 IMDs associated with various types of respiratory symptoms which were classified into seven groups according to the type of clinical manifestations affecting the respiratory system: (i) respiratory failure, (ii) restrictive lung disease, (iii) interstitial lung disease, (iv) lower airway disease, (v) upper airway obstruction, (vi) apnea, and (vii) other. We also provided a list of investigations to be performed based on the respiratory phenotypes and indicated the therapeutic strategies currently available for IMD-associated airway disease. This represents the thirteenth issue in a series of educational summaries providing a comprehensive and updated list of metabolic differential diagnoses according to system involvement.

## 1. Introduction

This is the thirteenth in a series of articles that aim to provide a comprehensive list of specific respiratory signs and symptoms associated with inherited metabolic disorders (IMDs). The first eleven issues were dedicated to IMDs associated with movement disorders [1], metabolic liver diseases [2], those with psychiatric presentations [3], metabolic cardiovascular diseases [4], those with cerebral palsy phenotypes [5], metabolic dermatoses [6], ocular phenotypes [7], neoplasms [8], metabolic ear diseases [9], metabolic myopathies [10], gastrointestinal symptoms [11] and immunological defects [12]. The list follows the classification of IMDs as included in the knowledge base of inborn errors of metabolism (IEMbase) [13], and in the Nosology of inborn errors of metabolism [14].

This issue is dedicated to respiratory disease which may occur in patients with IMDs.

## 2. Materials and methods

Source of the information was IEMbase the knowledgebase of IMDs (http://www.iembase.org) [15]. As of June 22, 2023, IEMbase tabulates 1878 IMDs and 4107 corresponding clinical and biochemical signs and symptoms grouped in 21 organ systems and conditions. Clinical symptoms associated with respiratory problems (*n* = 55) were extracted from the ‘Respiratory” group.

## 3. The respiratory system

Respiration is a vital process for the normal function at every level of organization from a cell to an organism. In humans, inhaled oxygen (O_2_) enables substrate oxidation in the mitochondria allowing for energy production. Carbon dioxide (CO_2_) generated by this process returns to the lung and eventually dissolves in the exhaled air. Two types of respiration are recognized: 1) cellular respiration (or oxidation); and 2) external respiration (or ventilation). External respiration requires integrity of upper and lower airways as well as, respiratory muscle force and cardiac function. It is also accurately regulated by central and peripheral nervous system. Dysfunction in any of these factors can result in respiratory signs and symptoms [16]. Hence, it is not surprising that respiratory manifestations are highly prevalent in patients with IMDs, in whom a multisystem involvement is commonly observed. Ventilation, perfusion and diffusion allow O_2_ supply and CO_2_ removal. During ventilation (V) intrathoracic airways expand in inspiration as intrapleural pressure becomes more negative and narrow in expiration as they return to baseline. Ventilation is achieved by the action of the respiratory muscles, which causes a change in the volume of the thoracic cage [17]. Inspiration involves contraction of the diaphragm and of the intercostal muscles, which expand the rib cage and may be supported by additional accessory muscles in case of respiratory distress. During normal expiration, a passive process takes place due to the elastic recoil of the lungs and surface tension, the diaphragm relaxes and goes up, while intercostal muscles relax and the rib cage collapses. Abdominal and external intercostal muscles are recruited in forceful expiration. Perfusion (Q) refers to the flow of blood to alveolar capillaries. The V/Q ratio evaluates the matching of alveolar V to Q. Changes in the V/Q ratio can contribute to hypoxemia. Gas exchange occurs in the alveoli by diffusion at the level of the alveolar capillary barrier. In diseases with abnormally increased alveolar capillary barrier e.g., interstitial lung disease (ILD) and lung fibrosis [18], gas diffusion gets impaired. As CO_2_ is much more diffusible than O_2_, in diseases characterized by diffusion defects significant increase of CO_2_ does not develop even in the presence of hypoxemia until hypoventilation also occurs.

## 4. Signs and symptoms

Childhood is a crucial period of lung development and early events to the airways can increase the risk of developing chronic airway disorders and even premature adult death [19]. The prevalence of airway disease in the general population is high, with a large proportion of children experiencing symptoms primarily due to infectious or atopic diseases [20]. As such, respiratory involvement can develop in patients with IMDs independent of the underlying disorder. On the other hand, many IMDs can be revealed by respiratory symptoms and, importantly, metabolic decompensation may be precipitated by airway infections.

Respiratory manifestations can occur either at presentation or in the late course of several IMDs, with variable degree of upper or lower airways and/or thoracic wall and/or neuromuscular involvement. Patients with IMDs can experience respiratory symptoms and signs that may not be easily attributed to the underlying disorder. For example, infants with biotinidase (BTD) deficiency may develop stridor not due to primary airway involvement but rather to vocal cord paralysis secondary to encephalomyelopathy [21].

The in-depth knowledge of the respiratory manifestations of IMDs would have two main benefits: i) earlier identification of IMDs and ii) better management of IMDs, thereby improving patients’ comfort and quality of life. Airway disease can result in frequent hospital admissions and can represent the ultimate reason of premature death. Therefore, establishing a diagnostic workup of the respiratory manifestations of IMDs is mandatory to improve the outcome of the underlying disorders. Finally, although many therapeutic strategies of IMDs are available, there currently exists no comprehensive review of the literature on the effects of IMD treatment on the airways to inform best daily practice.

**Fig. 1.**
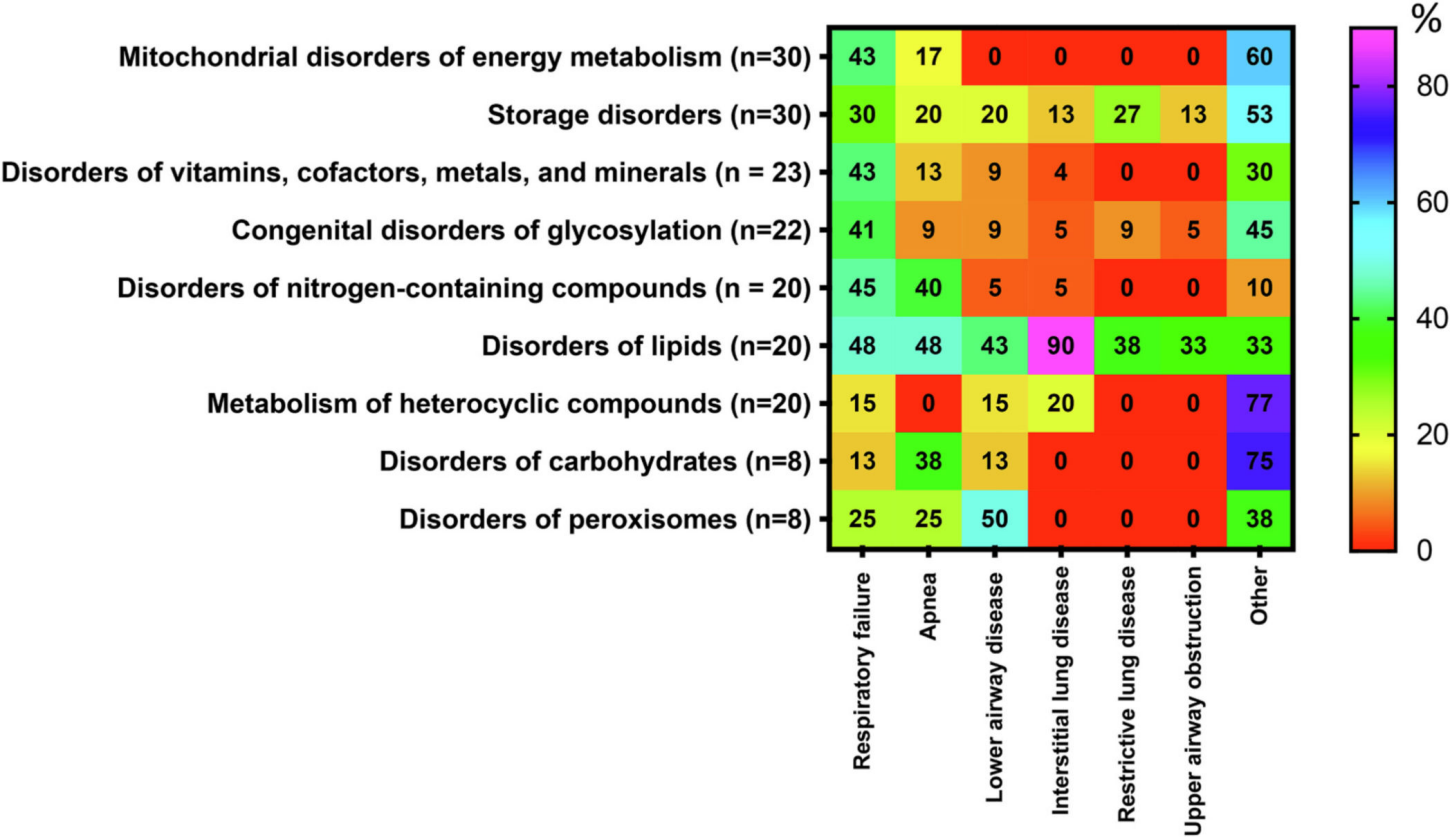
Occurrence (in percent) of respiratory symptoms associated with the categories of IMDs. The percentages for respiratory involvement were calculated using the denominator of the total number of IMDs in each category presenting with any respiratory phenotype. The heat scale ranges from red (0%; diseases with no particular symptom reported) to violet (100%; diseases with particular symptoms reported) within the disorders group. For further information about the 9 categories of disorders affecting respiratory system, see Supplemental Tables 1 and 2. For interpretation of references to colour in this figure legend, the reader is referred to the web version of this article.

We have identified 181 IMDs associated with different categories of respiratory symptoms. We classified the symptoms into seven groups according to the type of clinical manifestations affecting the respiratory system: (i) respiratory failure (including insufficiency), (ii) restrictive lung disease, (iii) interstitial lung disease, (iv) lower airway disease (including wheezing; bronchitis; recurrent pneumonia; aspiration pneumonia) (v) upper airway obstruction, (vi) apnea (including obstructive sleep apnea syndrome; sleep disordered breathing), (vii) other. The “other” category included recurrent respiratory infections (RRI), respiratory distress, pulmonary hypertension, pulmonary edema, pulmonary hemorrhages, inspiratory stridor, pulmonary hypoplasia, tachypnea, orthopnea, dyspnea, respiratory failure and diaphragm dysfunction, abnormal pulmonary lobation, laryngeal/tracheal calcification, underdeveloped lungs, aspiration, respiratory dysfunction, hypoplasia of the pharynx, tracheomalacia. The Supplemental Table 1 includes a list of all signs and symptoms included into the seven aforementioned groups, while Supplemental Table 2 sums up an exhaustive list of IMDs that can exhibit respiratory symptoms. Of all categories of respiratory symptoms, the most represented is the one including “other” types of symptoms (94/181; 52%), followed by respiratory failure (61/181; 34%), apnea (30/181; 17%), lower airway disease (22/181; 12%), interstitial lung disease (12/181; 7%), restrictive lung disease (10/181; 6%) and upper airway obstruction (6/181; 3%) (Fig. 1). Within the “other” category, the most frequent symptoms are RRI (25/94; 27%), pulmonary hypertension (23/94; 24%) and respiratory distress (22/94; 23%).

The group of IMDs most frequently associated with respiratory symptoms is mitochondrial disorders of energy metabolism (*n* = 30). Within this category, we particularly observed that disorders of complex IV subunits, disorders of the Krebs cycle, disorders of mitochondrial carriers, disorders of mitochondrial protein quality control and disorders of mitochondrial cofactor biosynthesis were mostly presenting with respiratory failure (13/30; 43%) and symptoms belonging to the “other” category (18/30; 60%), such as respiratory distress (7/18; 39%) and pulmonary hypertension (6/18; 33%).

Disorders of nitrogen-containing compounds were mostly associated with respiratory failure (9/20; 45%) and apnea (8/20; 40%).

Twenty-three different disorders of vitamins, cofactors, metals, and minerals displayed respiratory symptoms, in particular respiratory failure in 10/23 (43%) of cases.

Among disorders of carbohydrates, symptoms included in the “other” category were the most represented (6/8; 75%), such as tachypnea (3/6; 50%), RRI (2/6; 33%), pulmonary hypertension (2/6; 33%). Apnea (3/8; 38%), respiratory failure (1/8; 13%) and lower airway disease (1/8; 13%) were also detected.

Disorders of lipids were especially associated with a variety of respiratory symptoms, included in the “other” category (12/20; 60%), such as RRI (3/12; 25%) and respiratory distress (3/12; 25%) as well as respiratory failure (6/20; 30%).

Storage disorders displayed restrictive lung disease (8/30; 27%) and appeared to be particularly linked with respiratory failure (9/30; 30%). They were also found to be strongly associated with respiratory symptoms included in the “other” category, such as RRI (8/30; 27%).

Disorders of peroxisomes were associated with lower airway disease (4/8; 50%), apnea (2/8; 25%), RRI (2/8; 25%) and respiratory failure (2/8; 25%).

Congenital disorders of glycosylation have especially manifested respiratory failure (9/22; 41%); among symptoms included in the “other” category, the most frequent were respiratory distress (3/22; 14%) and RRI (3/22; 14%).

Disorders of metabolism of heterocyclic compounds were associated with “other” types of respiratory symptoms (17/20; 77%), especially pulmonary hypertension (8/20; 40%). In addition, in 3/20 (15%) disorders, respiratory failure was included in the clinical presentation of the disease.

Of all IMDs, specific subgroups are associated with well-defined airway conditions, for example, ILD or pulmonary hypertension or sleep-disordered breathing/obstructive sleep apnea syndrome (SDB/OSAS) or RRI (also due to airway aspiration) or, eventually, respiratory failure. In storage disorders (e.g., Niemann-Pick disease, Gaucher disease) and certain amino acid disorders (e.g., methylenetetrahydrofolate reductase [MTHFR] deficiency, lysinuric protein intolerance [LPI]) lower airway involvement may derive from accumulation of abnormal metabolites and/or macrophages loaded with storage material in the airspaces and interstitium, or from chronic airway aspiration associated with neuromotor delay [22–24]. Breathlessness, diminished exercise tolerance and impaired gas exchange result from the combined inflammation and fibrosis within the interstitial space [23,25]. Especially in cases who develop progressive pulmonary fibrosis, respiratory failure and death may ultimately occur [26]. Alveolar hypoventilation due to respiratory muscle dysfunction and/or abnormal respiratory mechanics related to enlarged abdominal organs is frequently reported in patients with storage disorders [2,27]. SDB due to craniofacial abnormalities and/or upper and middle airway soft tissue anomalies is a troublesome complication of several types of mucopolysaccharidoses (MPS; type I, II, IV, VI), especially in younger subjects [28,29]. Finally, pulmonary hypertension with chronic hypoxemia secondary to ILD or with intrapulmonary shunting secondary to liver disease may complicate some storage disorders [30–32]. Some IMDs with neuromuscular involvement can be associated with progressive chest and spine deformities, which predispose to RRI, chronic airway aspiration and progressive respiratory failure. These IMDs mainly include mitochondrial disorders, congenital disorders of glycosylation, peroxisomal disorders and amino acid disorders [33–37]. Immune dysfunction, such as chronic neutropenia or lymphocyte impairment, have been described in glycogen storage disease Ib and hereditary orotic aciduria and may explain the marked susceptibility to airway infections [38,39].

Since breathing is a dynamic process, the timing of airway manifestations may largely vary, with some already present at the time of IMD diagnosis and other occurring only at some stages of the disease. Indeed, some manifestations are age-dependent (e.g., RRI and OSAS) and may reflect what is observed in non-IMD individuals [40,41]. Hence, respiratory symptoms and signs in patients with IMDs should be regularly reassessed to optimize disease management.

## 5. Diagnosis

**Fig. 2.**
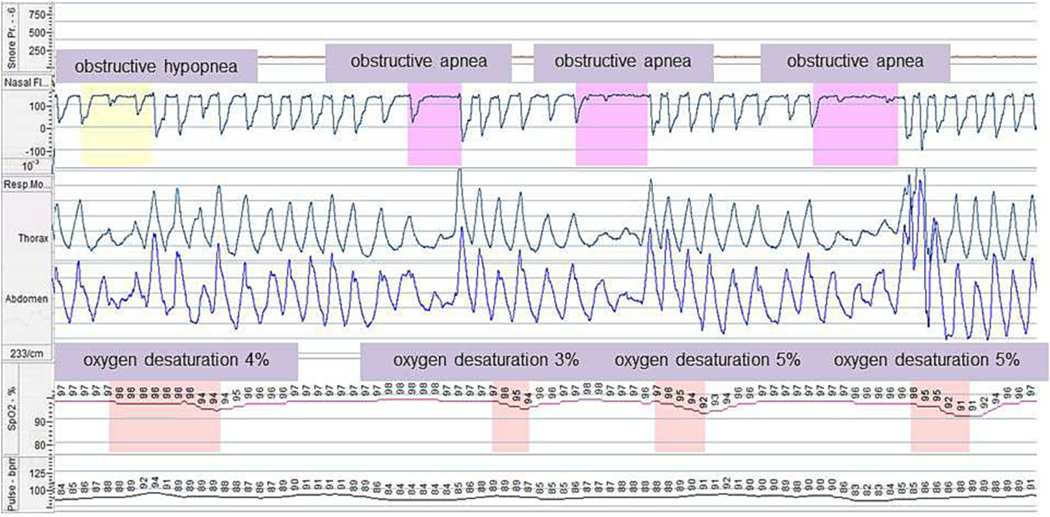
Respiratory polygraphy from a 5-year-old boy with Mucopolysaccharidosis type II. The cardiorespiratory monitoring used sensors to detect airflow (nasal air pressure transducer), respiratory effort (inductance plethysmography), transcutaneous blood oxygen (pulse oximetry) and pulse rate. This monitoring showed: obstructive apnea and hypopnea characterized by fall in amplitude of the nasal pressure > 90% and > 30%, respectively, and increase of respiratory effort with paradox; and oxygen desaturation >3%.

In the daily clinical practice, pulmonary function tests (PFTs) are largely used to diagnose lung disease. PFTs provide objective evidence of severity, help to monitor disease progression and treatment response by noninvasive determination of airflow mechanics and volumes (primarily by spirometry and plethysmography) [42], and of the diffusing capacity of the lungs for carbon monoxide (D_LCO)_ [43]. Spirometry is widely used at most hospitals for measuring the amount and/or speed (flows) of inhaled and exhaled air [43]. Lung volumes (dynamic or static) are assessed at different degrees of inspiration or expiration. The static lung volumes are distinguished into standard volumes and standard capacities. Dynamic volumes include forced vital capacity (FVC, the volume of air exhaled forcefully after maximal inspiration), forced volume exhaled in the first second (FEV_1_), and their ratio (FEV_1_/FVC). In restrictive lung disease, which can be due to pulmonary (such as ILD or lung fibrosis) or extrapulmonary causes (such as pleural diseases, obesity, neuromuscular disorders, and abnormalities of the rib cage or spine), volumes are reduced [42]. In obstructive diseases, characterized by distal airways narrowing as it occurs in wheezing disorders, reduction of FEV_1_ with respect to FVC and sometimes, but not always, of expiratory flow rates is reported at spirometry [42]. Values recorded at PFTs are compared with values derived from similar nonsmoking, race-, age- and gender-matched, disease-free subjects and are referred to as the “% of predicted value” [43]. In children, spirometry can be normal even in the presence of confirmed airway disease. In some cases, additional tests to evaluate airway resistance, D_LCO_, bronchodilator or exercise challenge tests may be necessary for defining lung processes but require more complex equipment and expertise. Although children can undergo office spirometry since age 5 years, lack of coordination in the respiratory maneuvers is a major barrier to PFTs [42]. Most employed tests of respiratory muscle strength include noninvasive measurements (such as maximum inspiratory/expiratory pressure) and in patients who can walk, the 6-min walk test (6MWT) which provides a measure of respiratory disability even in the presence of moderately severe impairment [44]. Transcutaneous O_2_ saturations (tcSpO_2_), as well as fatigue and dyspnea, are recorded during 6MWT. The 6MWT is widely used as single test at baseline and for assessing the response to therapeutic interventions. An abnormal 6MWT is nonspecific and non-diagnostic, thus additional tests investigating pulmonary or cardiac function, or muscular or bone/joint impairment are frequently required. At any age, even in the absence of patients’ cooperation, arterial blood gases (ABG) determination allows to assess respiratory function by measuring the partial pressure of O_2_ (PaO_2_) and of CO_2_ (PaCO_2_), and O_2_ saturation (SaO_2_) [45]. PaCO_2_ is closely related to depth and rate of breathing, thus it is a more sensitive marker of ventilatory failure than PaO_2_, particularly in the presence of supplemental O_2_ therapy. ABG provides insight on the degree or severity of abnormalities of the oxygenation and ventilation status, and clarifies whether these are acute or chronic, and if the primary disorder is respiratory in origin. Although ABG may be replaced by non-invasive monitoring, it is still useful in confirming results. Finally, the SDB entity, which includes the OSAS, the central sleep apnea, and the sleep-related hypoventilation/hypoxia, is a common cause of airway dysfunction in children [46] especially in cases with craniofacial alterations or soft tissues hypertrophy [47]. In patients with IMDs having symptoms or signs of upper airway obstruction [41], nocturnal polysomnography (PSG), or the alternate respiratory polygraphy, are widely used as objective, quantitative, non-invasive procedures of SDB [41] (Fig. 2).

**Fig. 3.**
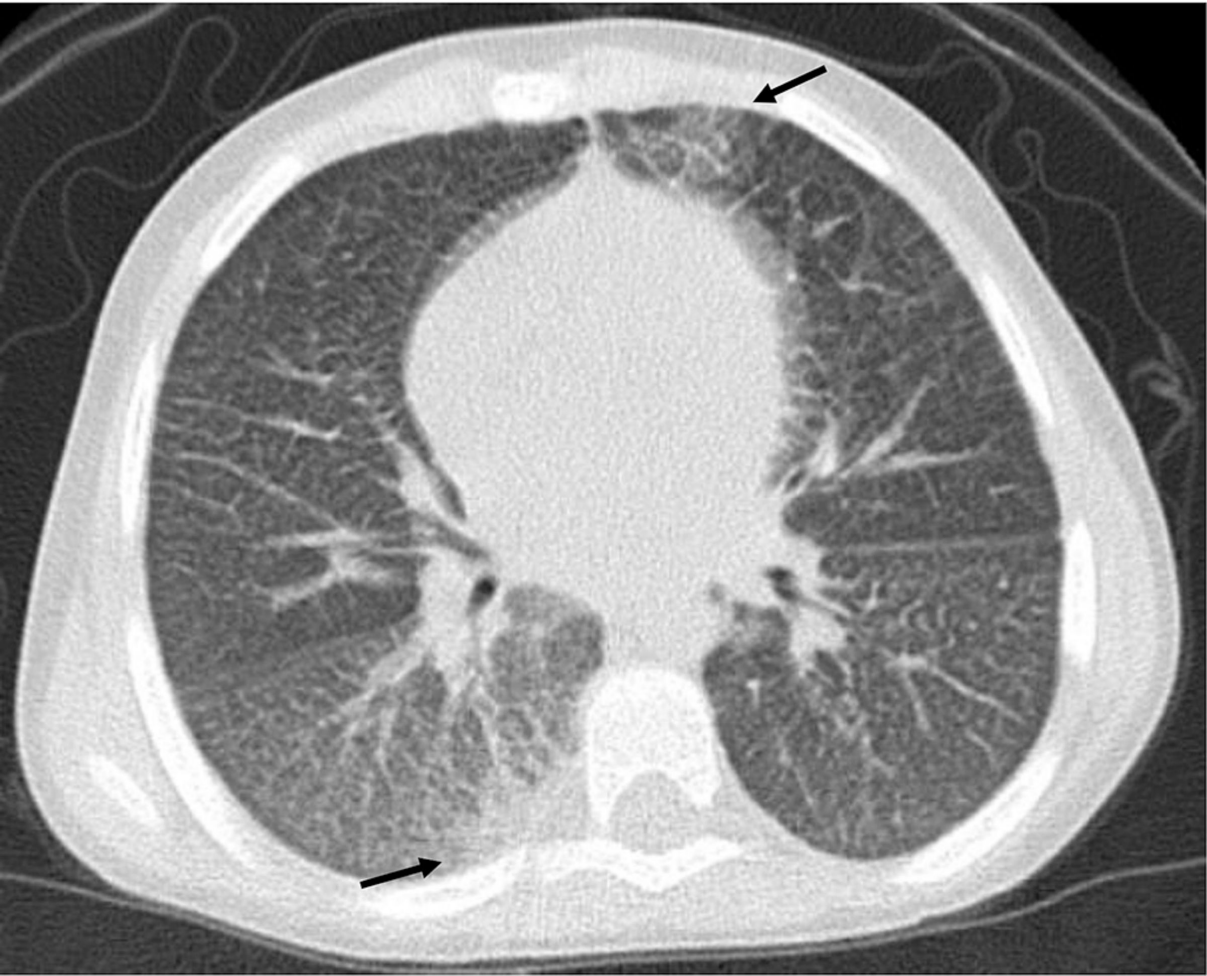
High resolution computed tomography scan of the chest from a 9-year-old boy with Niemann-Pick type B disease showing interstitial lung disease with bilateral diffuse severe thickening of the inter- and intralobular septa and focal areas of ground-glass opacity (arrows).

In addition to PFTs, the respiratory system can be assessed by means of various additional tests aimed at studying its structure, namely the imaging procedures and the endoscopy of the airways. Upper airways and chest imaging such as conventional X-rays and computerized tomographic (CT) scan are widely used at any age, The choice of the appropriate technique should start from the type of respiratory manifestation. High-resolution CT (HRCT) scan is preferred when a detailed study of lung parenchyma changes (nature and extension) is requested, especially those associated with ILD which may not be easily detected with a conventional chest X-ray [48] (Fig. 3). The risk of sedation and of high radiation exposure to children should not be neglected and be discussed. Other airway imaging techniques are radiation-free, including magnetic resonance imaging and ultrasonography of the airways, increasingly applied in neonatal as well as in pediatric patients despite their limitations [49,50]. Finally, in addition to the removal of foreign bodies and the demonstration of intrathoracic-extrapulmonary airway malformations, airway endoscopy has progressively gained more indications such as obtaining cytology and microbiology data from the bronchoalveolar lavage in cases with difficult-to-treat infectious or noninfectious lung disease [51].

Assessing airway impairment is mandatory in children with IMDs with respiratory manifestations, either in the presence or absence of overt respiratory compromise. A careful history including risk factors and features suggestive of respiratory manifestations and a thorough physical examination is key to increasing the likelihood of an accurate diagnosis. The list of major issues to be addressed at medical history must include i) the age at onset of symptoms and signs of airway manifestations; ii) the frequency and management of previous or current infections of either upper or lower respiratory tract, if any; iii) coughing or choking with foods, with focus on difficulties in clearing secretions; iv) daytime hypoventilation symptoms such as headache, nausea, tachycardia, sweating, fatigue and/or evidence of snoring, breathing effort and arousal (suggesting OSAS) and/or disturbed sleep, morning headache, morning anorexia or nausea, daytime sleepiness, fatigue, and poor concentration (suggesting nocturnal hypoventilation); v) any clues to muscle weakness and age at onset, also including degree of ambulation and muscle fatigability and its progression. At clinical examination vital signs, body weight, height, or ulna length/arm span must be recorded. Growth and nutritional status and finally, posture, seating and evidence of rib cage or spine deformities which might increase the risk of respiratory compromise should be assessed.

When planning investigations, a detailed knowledge of the pathophysiological mechanisms underlying respiratory impairment is imperative [52]. Major respiratory investigations in children with IMDs must be established starting from the different categories of the associated respiratory symptoms (Table 1). The agenda includes an initial assessment *plus* close follow-up visits scheduled according to the evolution of the underlying disorder, with special attention to patients at risk for metabolic decompensation. In addition to pediatric pulmonologists, the team should include other specialists, namely radiologists for chest and spinal imaging, otolaryngologists for the upper airways, orthopedists for spinal deformities, and gastroenterologists for the assessment of swallowing disturbances. The consultation of a pediatric anesthesiologist may be requested in IMD patients with recognized difficult airways, such as MPS [53].

## 6. Treatment

### 6.1. General treatments

In patients with IMDs at risk of airway disease, specific therapies targeted at encouraging and restoring the physiology of the airways are strongly suggested since the earliest stages of the disease. The aims are to clear the airways, to prevent infections, and to maintain adequate alveolar ventilation. The choice of any therapy should be made on an individual basis after considering patient’s age and severity of the manifestation. Upper and/or lower airways disease may require inhaled and/or systemic steroids and/or inhaled bronchodilators for treating local inflammation and relieving bronchial obstruction, respectively [54]. Daily and/or nocturnal O_2_ supplementation is mandatory in cases with abnormal gas exchange. In cases with recurrent and/or aspiration pneumonia the cornerstones of treatment are mobilization of secretions (through individualized chest physiotherapy), and prevention and treatment of airway infections. Antibiotics should be preferably administered according to sputum or deep throat culture. In certain patients, especially those with respiratory insufficiency and/or upper airway obstruction with SDB or documented OSAS, invasive or noninvasive mechanical ventilation (e.g., continuous positive airway pressure, bilevel positive airway pressure) may be necessary [55,56]. However, adherence or acceptance of the device can importantly limit its use, especially in cases with neurological impairment. Upper airway obstruction may also benefit from adenotonsillectomy even though difficulties in intubation are frequently reported in patients with MPS because of thickened and stiffened tissues in the laryngopharynx and trachea [53].

### 6.2. Specific treatments

Over the past years, specific therapeutic strategies have been developed for various IMDs. Different options have become available in clinical care, including enzyme replacement therapy (ERT), substrate reduction therapy (SRT), and hematopoietic stem cell transplantation (HSCT) which may exert a beneficial effect also on the associated respiratory manifestations [57]. In many IMDs, acute respiratory symptoms can be triggered by metabolic decompensation; at the same time, respiratory infections can trigger metabolic decompensation. Therefore, an appropriate emergency plan to prevent catabolism should be rapidly put in place [58]. The National Immunization Schedule, the annual influenza vaccine as well as the antipneumococcal vaccine are strongly recommended. Only in IMD patients with an altered immune response, vaccinations with live attenuated vaccines are not recommended [59]. The recent severe SARS-CoV-2 pandemic critically affected many fragile patients. Surprisingly, the European Reference Network for Hereditary Metabolic Diseases (MetabERN) reported that the incidence of COVID-19 disease in patients with IMDs was lower than in the general European population [60]. Although most patients presented with mild symptoms, some fatal events were recorded [61]. Overall, considering the limited knowledge on the long-term effects of COVID-19 and the potential risk of metabolic decompensation, experts suggest that IMD patients should be vaccinated against SARS-CoV-2, even though the topic is still debated [60,61]. Specific treatment options for respiratory manifestations in major IMDs are discussed below.

#### 6.2.1. Disorders of nitrogen-containing compounds

In methylmalonic aciduria (MMA), propionic aciduria (PA), maple syrup urine disease (MSUD) and glutaric aciduria type I (GA I) respiratory manifestations result from the combination of metabolic decompensation, cardiomyopathy and/or neuromuscular involvement. As such, low-protein diet as well as long-term pharmacotherapy [62–67] can decrease the risk of decompensation-related respiratory manifestations. Although liver transplantation may improve both cardiomyopathy and neurological symptoms [68,69] the effect on respiratory manifestations has not been systematically assessed.

In non-ketotic hyperglycinemia (NKH) combination of sodium benzoate with *N*-methyl-d-aspartate (NMDA) receptor site antagonists (e.g., dextromethorphan, ketamine) may improve apnea and hypotonia [70]. Yet, the clinical response may vary between i) severe and attenuated NKH and ii) early and late disease stage. Overall, the long-term patient outcome remains poor [71].

Pulmonary alveolar proteinosis (PAP) is a progressive severe syndrome which may complicate the course of LPI in up 75% of cases [72,73]. Patients may present either with poorly symptomatic lung disease or even progressive breathlessness and irreversible respiratory failure. Treatment of lung disease in LPI remains controversial since high-dose steroid treatment was beneficial in a few patients when started early, whereas no response was noted in others [73,74]. Administration of granulocyte/monocyte colony-stimulating factor (GM-CSF) was shown to be ineffective or even to worsen the clinical course in one case [75]. However, in a Finnish study GM-CSF appeared to benefit two individuals with severe PAP [75]. Whole-lung lavage remains the best therapeutic approach for PAP in LPI although relapses may require serial lavages [76]. Heart-lung transplantation was attempted with a temporary successful result but did not prevent a fatal return of lung disease [77]. HSCT has been discussed as a possible treatment for PAP in LPI to correct the defective function of lung macrophages, but currently no experience has been reported [7,78,79].

#### 6.2.2. Disorders of vitamins, cofactors, metals and minerals

A few patients with methylmalonic aciduria and homocystinuria cblC type have been reported who developed late-onset diffuse lung diseases and pulmonary arterial hypertension [80]. Similar to disorders of nitrogen-containing compounds, treatment of the underlying disorder together with sildenafil improved respiratory symptoms in most patients [80].

In BTD deficiency, respiratory manifestations are prevented by early oral biotin supplementation if treatment starts before irreversible neurological damage occurs. Late-diagnosed patients often have neurological involvement, thus requiring respiratory supportive care [81].

Prompt institution of appropriate therapy (i.e., betaine, folinic acid) can allow complete resolution of respiratory symptoms (apnea, respiratory failure) in MTHFR deficiency [82]. Late-diagnosed individuals present with neurocognitive involvement and apnea which may require respiratory supportive care.

In most patients with riboflavin transporter deficiency, pulmonary function and muscle strength improve upon riboflavin supplementation provided treatment is started early [83].

Although replacement therapy with fosdenopterin has been shown to decrease the risk of death and improve neuromotor function in molybdneum cofactor deficiency type A, respiratory outcomes have not been systematically assessed. However, fosdenopterin must be initiated in a very short window after manifestations of symptoms to have maximum therapeutic benefit. Long-term efficacy remains to be investigated [84].

#### 6.2.3. Disorders of carbohydrate metabolism

In fructose-1,6-bisphosphatase deficiency, GSDIa and GSDIb, hyperventilation develops during metabolic decompensation. As such, dietary treatment (i.e., frequent feedings, uncooked cornstarch and/or gastric-drip feeding) is aimed at preventing decompensation. G-CSF is indicated in GSDIb to prevent RRI [85]. One patient with GSDIa and pulmonary hypertension was successfully treated with sildenafil [86].

#### 6.2.4. Storage disorders

In Gaucher disease (GD), ERT was shown to improve lung expansion by decreasing the risk of the hepatopulmonary syndrome [87]. However, the effect of ERT in preventing and/or reversing lung disease is unclear. ERT was shown indeed to be poorly effective on pulmonary hypertension and ineffective at all on lung fibrosis [88], probably because of its poor penetration into the lungs [87]. In patients with severe lung involvement and suboptimal response to ERT, HSCT could be beneficial [88]. In contrast to ERT, SRT (i.e., miglustat, eliglustat) allows a greater tissue penetration [89]. Improved pulmonary function was observed in patients with type III GD who did not respond to ERT [90]. Successful experience with various vasodilators (namely, prostacyclin, sildenafil) has been reported in the treatment of pulmonary hypertension in GD [91]. Eventually, GD patients experiencing ILD and/or pulmonary hypertension may require lung transplantation [92]. Improvement in lung function has been reported provided that ERT is continued in the post-transplant course. Yet, experience remains limited [93,94].

There is controversial evidence on the effects of ERT on pulmonary symptoms in Fabry disease [95]. However, treatment with ERT resulted in a beneficial effect on pulmonary function in various patients’ cohorts irrespective of their genotype [96–100].

In Niemann-Pick type A and B (NPA and NPB) disease, an improvement of ILD-related HRCT findings was observed in patients receiving ERT, although data is still scant [101,102]. SRT can prevent or improve dysphagia and airway aspiration in Niemann-Pick type C (NPC) disease [103]. HSCT has been shown to favor the regression of lung infiltrates at chest imaging. However, lack of stabilization of the neurologic involvement, together with HSCT high morbidity and mortality greatly limit its use [104]. Administration of GM-CSF was shown to be effective in one patient with NPB [105] and ineffective in one patient with NPC who developed PAP [106]. Half-lung lavage was reported to be only temporarily effective on PAP in NPC [107]. In Pompe disease (PD) ERT seems to stabilize or even improve pulmonary function [107] decreasing the need of ventilation support [108]. Significant improvement of the 6MWT and of pulmonary function was observed even in ventilator-dependent patients treated with ERT [109,110]. ERT slows progression of respiratory disease and improves upper airway obstruction and sleep apnea in MPS I [111], pulmonary function in MPS II [112] and the 6MWT in a cohort of MPS IVA patients [113]. Conversely, the effect of ERT on respiratory manifestations in MPS VI remains to be ascertained [114]. Although HSCT can slow the progression of respiratory symptoms, patients may still develop hypoxia and eventually need ventilation support [115]. Combined HSCT and ERT have been associated with improved pulmonary function in one patient with MPS VI [116].

#### 6.2.5. Disorders of peroxisomes

Respiratory symptoms result from neurological involvement in X-linked adrenoleukodystrophy (X-ALD) and Zellweger syndrome. In X-ALD, HSCT can arrest cerebral demyelination when treatment is made before the onset of early symptoms [117]. Yet, respiratory outcomes have not been systematically assessed. No improvement in clinically relevant parameters was observed in a cohort of patients with Zellweger syndrome treated with oral cholic acid [118].

#### 6.2.6. Innovative treatments

**Fig. 4.**
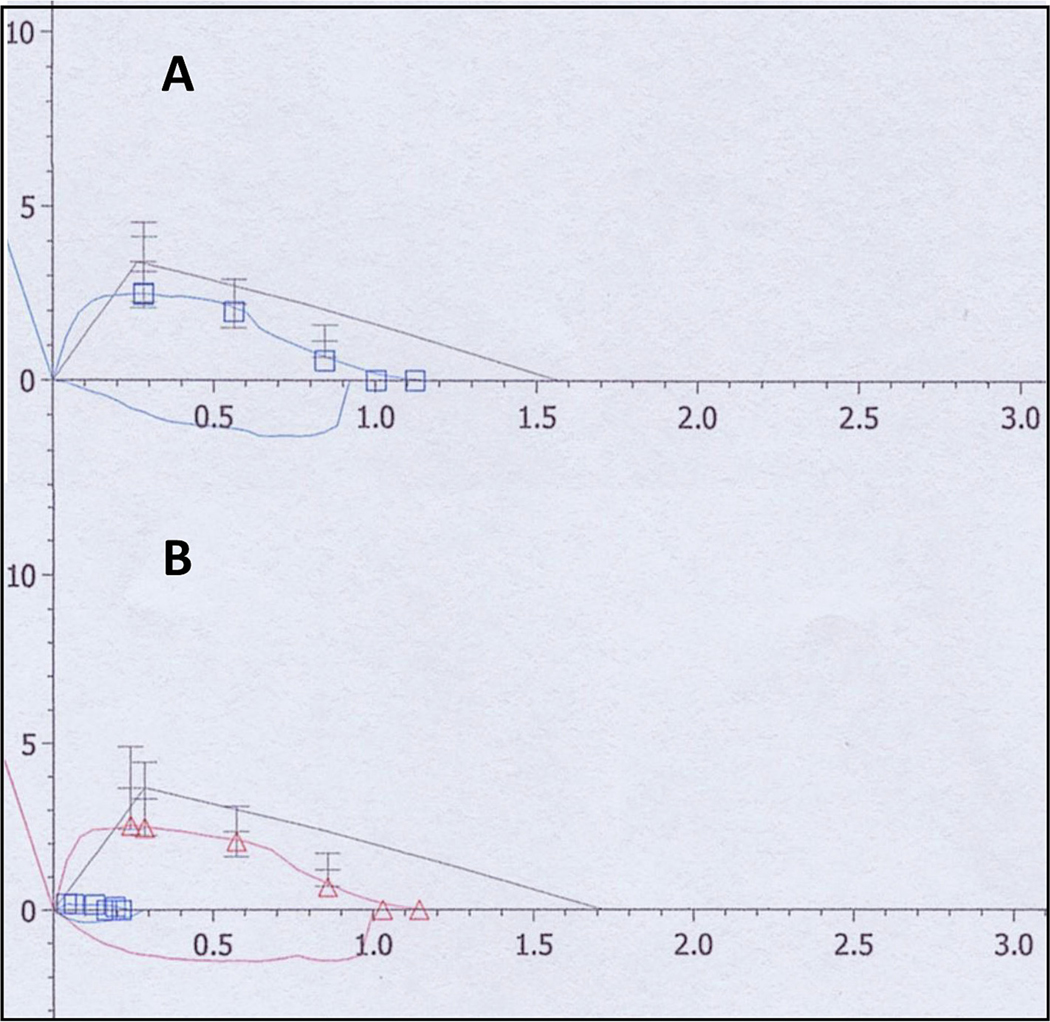
Flow/volume curve from a 10-year-old girl with Mucopolysaccharidosis type VI. Spirometry made before (A; blue tracing) and 4 months after (B; red tracing) liver-directed gene therapy (and after ERT discontinuation) show substantially unchanged FVC (1.12 versus 1.14 l, respectively), FEV_1_ (1.00 versus 1.03 l, respectively) and forced expiratory flows at 75% of exhaled FVC (2.46 versus 2.46 l, respectively). (For interpretation of the references to colour in this figure legend, the reader is referred to the web version of this article.)

In addition to currently available therapies, novel treatment options (e.g., gene therapy, mRNA therapy, pharmacological chaperones, drug repurposing) which have the potential to be effective on the respiratory manifestations in IMDs are also being investigated. Improvement in FVC was observed in a cohort of patients with late-onset PD treated with ERT/miglustat (a pharmacological chaperone) as compared to patients receiving ERT alone [119]. Liver-directed gene therapy was shown to stabilize pulmonary function in a subset of patients with MPSVI who discontinued ERT [120] (Fig. 4). Treatment with empagliflozin (SGLT-2 inhibitor) was associated with increased neutrophil count and decreased neutrophil dysfunction–related symptoms in a cohort of patients with GSDIb [121]. Moreover, in utero ERT is being explored in a group of lysosomal storage disorders (NCT04532047), with potential to prevent storage-related respiratory manifestations. Information on clinical trials on innovative treatments for IMDs can be found at https://clinicaltrials.gov/ and https://www.clinicaltrialsregister.eu/ctr-search/search.

## 7. Conclusion

Extensive knowledge of the respiratory phenotypes occurring in patients with IMDs allows early recognition and treatment of these manifestations. We provided a comprehensive list of IMDs associated with respiratory manifestations, and proposed a list of investigations to be performed based on the respiratory phenotypes as well as available treatment options.

Despite the progress occurred over the past decades, the management of respiratory manifestations in IMDs is still suboptimal. Of all current treatment options, many are ineffective or only partially effective because are untargeted to the underlying enzyme defect or do not sufficiently penetrate the airways. Thus, respiratory disease poses a major burden on patients’ prognosis as it significantly affects morbidity or mortality, and irreversible respiratory failure may eventually develop in many patients. Similarly, current monitoring methods are unsatisfactory in predicting patients’ outcome or cannot be performed because require patients’ collaboration or are invasive. The development of novel monitoring and treatment options is expected to address the compelling need for improved management options to optimize patient outcome.

This represents the 13th issue in a series of educational summaries providing a comprehensive and updated list of metabolic differential diagnoses according to system involvement. The full list can be freely accessed at www.iembase.org/gamuts and will be curated and updated on a regular basis. Supplementary data to this article can be found online.

## Supplementary Material

1

2

## Figures and Tables

**Table 1:** Major investigations of respiratory manifestations in children with inherited metabolic diseases.

Respiratory manifestation	Procedure

Upper airways obstruction	Upper airways imaging Spirometry^a^ Airway endoscopy Microbiological and/or virological tests on airway sample^b^ Arterial blood gases
Apnea	Overnight sleep monitoring (pulse oximetry; polysomnography/polygraphy; tcCO_2_) Arterial blood gases
Lower airway disease	Chest imaging Spirometry; Plethysmography Airway endoscopy Microbiological and/or virological tests on airway sample^b^ Arterial blood gases Swallowing/aspiration studies^c^
Interstitial lung disease	Chest imaging Airway endoscopy and bronchoalveolar lavage Spirometry; Plethysmography; D_LCO_ Arterial blood gases
Restrictive lung disease	Chest imaging Spirometry; Plethysmography; D_LCO_ Arterial blood gases Spine imaging
Respiratory failure	Arterial blood gases Pulse oximetry (daytime/overnight) tcCO_2_ monitoring (daytime/overnight) Polysomnography/polygraphy Chest imaging
Other (RRI; respiratory distress; pulmonary hypertension; pulmonary hypoplasia; underdeveloped lung; pulmonary edema; dyspnea; hypoplasia of the pharynx; tracheomalacia)	Chest imaging Arterial blood gases Microbiological and/or virological tests on airway sample^b^ Immune studies Cardiac assessment Airway endoscopy

Abbreviations: D_LCO_, diffusing capacity of the lungs for carbon monoxide; tc, transcutaneous; CO_2_, carbon dioxide; RRI, recurrent respiratory infections.

a In cooperating patients; impulse oscillometry in noncooperating patients.

b Sputum or deep oropharyngeal aspirate or nasopharyngeal swab.

c Including video fluoroscopy swallow study; fiberoptic endoscopic evaluation of swallowing.

## Data Availability

Data will be made available on request.
