# Supplementary material for: Clinical and biochemical footprints of inherited metabolic diseases. XIII. Respiratory manifestations
Source: Mol Genet Metab. Author manuscript; Available in PMC 2025 Jan 22. (PMC11753447; doi:10.1016/j.ymgme.2023.107655)
Supplement: 1 [file NIHMS2044629-supplement-1.docx]

**Supplementary Table 1.** Clinical terms reported in inherited metabolic diseases with respiratory manifestation.

| **Respiratory failure (including insufficiency)** |
| --- |
| Cardiopulmonary failure |
| Dyspnea |
| Hypoxia |
| Orthopnea |
| Pulmonary insufficiency |
| Respiratory failure |
| **Restrictive lung disease** |
| Breathing difficulty |
| Respiratory distress |
| Respiratory dysfunction |
| Respiratory insufficiency |
| Restrictive lung disease |
| **Interstitial lung disease** |
| Chest radiographs, Interstitial changes |
| Interstitial lung disease |
| Interstitial pneumonitis |
| Pulmonary alveolar proteinosis |
| Pulmonary interstitial changes |
| Respiratory bronchiolitis |
| **Lower airway disease (including wheezing; bronchitis; recurrent pneumonia; aspiration pneumonia)** |
| Alveolar capillary dysplasia |
| Broad alveolar ridges |
| Hemopneumothorax |
| Hypolobated lungs |
| Lung bleeding |
| Lung infiltrates |
| Pneumonia |
| Pneumothorax |
| Recurrent bronchopneumonia |
|  |
| **Upper airway obstruction** |
| Hoarseness |
| Nasal congestion |
| Snoring |
| Stridor, inspiratory |
| **Apnea (Including obstructive sleep apnea syndrome; sleep disordered breathing)** |
| Apnea |
| Hypoventilation, central |
| Obstructive sleep apnoea |
| Upper airway obstruction |
| **Other** |
| Airway obstruction |
| Asthma |
| Bronchiectasis |
| Diaphragm dysfunction |
| Laryngeal calcification |
| Nasal septum perforation |
| Profuse nasal secretion |
| Pulmonary edema |
| Pulmonary fibrosis |
| Pulmonary hypertension |
| Pulmonary hypoplasia |
| Pulmonary lobation, abnormal |
| Pulmonary stenosis |
| Punctate calcifications of larynx |
| Punctate calcifications of trachea |
| Small and non-foamy alveolar macrophages at bronchoalveolar lavage |
| Tachypnea/polypnea (e.g Kussmaul breathing) |
| Tracheal calcification |
| Underdeveloped lungs |
| Unilobular lung |
